# Novel Calcium Phosphate Promotes Interbody Bony Fusion in a Porcine Anterior Cervical Discectomy and Fusion Model

**DOI:** 10.1097/BRS.0000000000004916

**Published:** 2024-01-12

**Authors:** Maria Östman, Peter Försth, Patricia Hedenqvist, Håkan Engqvist, Leticia Marcelino, Bjørnar Ytrehus, Gry Hulsart-Billström, Michael Pujari-Palmer, Caroline Öhman-Mägi, Odd Höglund, Franck Forterre

**Affiliations:** a Department of Clinical Veterinary Medicine, Division of Small Animal Surgery, Vetsuisse Faculty, University of Bern, Bern, Switzerland; b Department of Surgical Sciences, Division of Orthopedics, Uppsala University, Uppsala, Sweden; c Department of Clinical Sciences, Swedish University of Agricultural Sciences, Uppsala, Sweden; d Department of Materials Science and Engineering, Division of Applied Materials Science, Uppsala University, Uppsala, Sweden; e University Animal Hospital, Swedish University of Agricultural Sciences, Uppsala, Sweden; f Department of Biomedical Sciences and Veterinary Public Health, Swedish University of Agricultural Sciences, Uppsala, Sweden; g Norwegian Veterinary Institute, Ås, Norway; h Department of Medicinal Chemistry, Science for Life Laboratory, Uppsala University, Uppsala, Sweden

**Keywords:** anterior cervical fusion, pseudarthrosis, interbody fusion, pig, bone, synthetic bone graft, monetite, calcium pyrophosphate, computed tomography, micro-CT, histopathology

## Abstract

**Study Design.:**

Experimental porcine anterior cervical discectomy and fusion (ACDF) model: a proof-of-concept study.

**Objective.:**

The effect of monetite synthetic bone graft (SBG) containing calcium pyrophosphate and β-tricalcium phosphate on cervical spinal fusion in a noninstrumented two-level large animal model.

**Summary of Background Data.:**

ACDF is the gold standard surgical technique for the treatment of degenerative cervical spinal diseases. However, pseudarthrosis associated with increased patient morbidity occurs in ∼2.6% of the surgeries. SBG may enhance bony fusion and subsequently decrease the risk of pseudarthrosis. Recent studies on monetite-based SBGs for use in large cranial defects in humans have shown promising bone healing results, necessitating further investigation of their use in cervical spinal fusion.

**Materials and Methods.:**

Four adult female Danish Göttingen minipigs received partial cervical anterior discectomy and intervertebral defects at an upper and lower level. One defect was filled with SBG, and the other was left empty. Bony fusion was evaluated using computed tomography (CT) at three-month intervals for 12 months. Fifteen months postsurgery, the animals were euthanized for further *ex vivo* qualitative histopathologic and micro-CT evaluations. Fusion rates were compared using the Fisher exact test at each time point.

**Results.:**

Increased interbody bony fusion rates were observed at SBG levels (4/4) compared with control levels (0/4) evaluated by CT at 6 and 9 months postsurgery (*P*=0.029). Fusion was observed at all SBG levels 12 months postsurgery and at only one control level. Histopathologic evaluation confirmed high-quality interbody bony fusion at all SBG levels and fusion by spondylosis at one control level.

**Conclusion.:**

This proof-of-concept study provides preliminary evidence of a novel, calcium pyrophosphate-containing, and β-tricalcium phosphate-containing monetite SBG that promotes bony fusion compared with a negative control in a clinically relevant porcine model of ACDF.

Degenerative cervical spine disease is a global health concern and is associated with a substantial socioeconomic burden and patient morbidity.^1,2^ To treat degenerative cervical conditions, anterior cervical discectomy and fusion (ACDF) is the gold standard surgical technique.^3–5^ In the United States, anterior fusion procedures accounted for 80.6% of all cervical spine surgeries from 2001 to 2013, and around 127,500 ACDF surgeries were performed in 2013. ACDF is considered one of the most costly surgical procedures in the United States, accounting for a total of 2.152 billion yearly in aggregated costs.^6^ This procedure typically results in excellent clinical outcomes. However, in 2.6% of the surgeries, failure of interbody bony union results in pseudarthrosis.^7^ This outcome is associated with increased patient morbidity, for example, persistent neck pain, instability, radiculopathy, myelopathy^8^ and is a leading cause of revision surgery associated with poorer quality of life.^9^ Reoperating rates have been reported to range between 0.58% and 11.1% depending on the number of levels involved.^10–13^ Autologous bone grafting (ABG) is used to assist bony fusion and thereby avoid pseudarthrosis.^14^ Synthetic alternatives, such as calcium phosphate-based grafts (CaPBGs), are often used instead of ABG. In comparison, they are simple to use and decrease surgery time and unnecessary donor site morbidity.^15–17^ Several CaPBGs have excellent osteoconductive and degradation properties, as well as biocompatibility.^18,19^ Some recently developed multicomponent CaPBGs have shown osteoinductive behavior ascribed to incorporating different phases of calcium phosphate (CaP), such as monetite, or both beta-tricalcium phosphate (β-TCP) and calcium pyrophosphate (Ca-PP).^20–23^ Recent studies on a monetite-based synthetic bone graft (SBG) have shown promising bone healing results in the treatment of large cranial defects in humans.^24–27^ The present proof-of-concept study, therefore, aimed to evaluate a novel Ca-PP-containing and β-TCP-containing monetite SBG in comparison to control level surgery in a noninstrumented two-level ACDF porcine model. It was hypothesized that SBG would promote interbody cervical spinal fusion in comparison with the negative control level in this clinically relevant porcine model of ACDF.

## MATERIALS AND METHODS

### Study Design and Animal Model

The proof-of-concept study was approved by the Animal Ethics Board in Uppsala, Sweden (protocol:08751-2019) and conducted under Good Laboratory Practices at the University of Agricultural Sciences in Uppsala, Sweden, between November 2019 and February 2021 in accordance with the Swedish National Guidelines for the Care and Use of Laboratory Animals.^28^ Four separately housed adult female Danish Göttingen minipigs (N4; Ellegaard Göttingen Minipigs, Denmark; see Table, Supplemental Digital Content 1, http://links.lww.com/BRS/C369, experimental animals) received anterior partial discectomy and intervertebral defect at an upper and lower level of the cervical vertebral column. One defect was randomly allocated to be filled with SBG, whereas the other defect was left empty as an internal negative control. The primary outcome was bony fusion, evaluated using computed tomography (CT) every three months, over 12 months. Secondary imaging outcomes were evaluated at the same time intervals. Fifteen months postsurgery, the animals were euthanized (Table, SDC2, euthanasia, Supplemental Digital Content 2, http://links.lww.com/BRS/C370) for further *ex vivo* qualitative histopathologic and μCT evaluations.

### Preparation of SBG

Dicalcium phosphate dihydrate was prepared from a mixture of the two precursors. The first precursor consisted of β-TCP containing 10 wt% β-calcium pyrophosphate (β-CPP) synthesized as described below, whereas the second precursor consisted of monocalcium phosphate monohydrate (MCPM, Scharlau) sieved below 75 μm, mixed at a β-TCP:MCPM molar ratio of 1:0.818, and then mixed with water at a ratio of 0.22 mL/g. Specimens were injected into molds, allowed to react for 24 hours, and then sterilized by autoclaving at 121°C for 40 minutes and the following material composition was yielded: 1% dicalcium phosphate dihydrate, 9% β-CPP, 6% β-TCP, 1% MCPM, and 83% dicalcium phosphate anhydrous, as determined from X-ray diffraction and Rietveld refinement.

### Surgical Procedure

In short, using an anterior median approach to the cervical spine, intervertebral spaces were exposed at an upper level of C2/3 or C3/4 and a lower level of C4/5 or C5/6 with an intermediate level left intact. Two defects, ranging between ∼172 and 945 mm^3^, were created for each animal by resecting parts of the anterior aspect of the exposed disc tissue, end plates, and vertebral bodies, with the dorsal longitudinal ligament remaining intact. Thereafter, the defects were filled with SBG, morselized using forceps, or left empty. No vertebral fixation was used. Routine closure was performed. Surgeries were performed under general anesthesia with perioperative and postoperative analgesia (Table, SDC2, anesthesia and analgesia, Supplemental Digital Content 2, http://links.lww.com/BRS/C370) by the same board-certified veterinary surgeon, intending to create a similar defect size at each operated level. The animals were monitored continuously for seven days postsurgery using video surveillance as part of another study.

### Computed Tomography

The cervical spine was scanned at an isotropic resolution of 0.6 mm (Somatom Definition AS 64; Siemens, The Netherlands). CT 3D multiplane reconstruction was performed with 0.6 mm slice thickness and a B70s kernel of the spine helical series with a voxel size of ∼0.25×0.25×0.6 mm. CT analysis was performed according to a protocol (Figure, SDC 1-5, CT protocols, Supplemental Digital Content 3, http://links.lww.com/BRS/C371, Supplemental Digital Content 4, http://links.lww.com/BRS/C372, Supplemental Digital Content 5, http://links.lww.com/BRS/C373, Supplemental Digital Content 6, http://links.lww.com/BRS/C374, Supplemental Digital Content 7, http://links.lww.com/BRS/C375) prepared by a board-certified veterinary radiologist and analyzed in a nonblinded manner by a supervised veterinary imaging resident. Fusion was evaluated as interbody bony fusion (IBF) and fusion by spondylosis (FbS), defined as any continuous trabecular bony bridge within the disc space or outside the disc space, respectively. Fusion was classified binary as “fused” or “nonfused.” Intervertebral disc space was measured at four different anatomical locations: anterior, posterior, right, and left at the midsagittal section and normalized to baseline intervertebral disc space. The degree of sclerosis, vertebral body lysis, and bone formation within the disc space were also recorded. To estimate the amount of anterior bone formation, the spondylosis anteroposterior dimension ratio (R_ab_) was calculated. Horos software (Nimble Co LLC d/b/a Purview Annapolis, MD, USA) was used for image analysis and processing.^29^

### Microcomputed tomography

After euthanasia of the animals 15 months postsurgery, the cervical vertebrae were examined with micro-CT (μCT; SkyScan 1176, Kontich, Belgium) at 90 kV voltage and 278 µA current, using a 0.1 mm Cu filter and 360° scan with a voxel size of 9 μm, ∼80x the resolution of traditional CT. Images were reconstructed with NRecon and visualized using the CTVox software (SkyScan; Bruker MicroCT). Fusion was evaluated at the control and SBG levels using the same definition as that used for CT evaluation.

### Histopathology

Necropsy was performed on all animals, and routine histology of tissues from the heart, lung, liver, kidney, and fundus of the ventricle was performed. The tissue was fixed in 10% phosphate-buffered formalin, stained with hematoxylin and eosin (kidney sections were also stained with van Gieson’s), and examined under a light microscope for histopathologic lesions according to standard procedures. Following μCT, undecalcified histologic processing was performed on vertebral column specimens. Vertebral columns were fixed in 10% phosphate-buffered formalin for seven days and stored in 70% ethanol for approximately seven months at room temperature until they were processed for histopathology. Segments were cut using an oscillating saw in the sagittal plane. Each specimen was then dehydrated in graded solutions of alcohol and infiltrated with increasing concentrations of polymethylmethacrylate (Technovit 7200 Kulzer GmbH, Hanau, Germany) mixed with 99.9% ethanol before being polymerized into hardened polymethylmethacrylate blocks. Sections were produced in the sagittal plane at a thickness ranging between 16 and 64 µm and stained with modified paragon stain for histopathologic examination. The left, central, and right sagittal sections were examined under a light microscope and imaged using a microscope camera. Fusion was evaluated in all sections, and the local tissue response to the material was scored semiquantitatively (Table, SDC3, scoring scheme, Supplemental Digital Content 8, http://links.lww.com/BRS/C376) according to the International Organization of Standardization (10993-6:2016)^30^ by a nonblinded, experienced veterinary pathologist.

### Statistical Analyses

Statistical analyses were performed on all outcome parameters using GraphPad Prism version 9 (Boston, Massachusetts, USA). The median values with the minimum and maximum values are presented (range). Fusion rates were compared using the Fisher exact test for each timepoint; intervertebral disc space and R_ab_ using multiple Wilcoxon tests corrected for multiple comparisons. Statistical significance was set at *P*<0.05.

## RESULTS

No intraoperative or major postoperative complications were noted. Necropsy revealed no major pathology (Figure, SDC6, summary of the necropsy, Supplemental Digital Content 9, http://links.lww.com/BRS/C377).

### Computed Tomography

**Figure 1:**
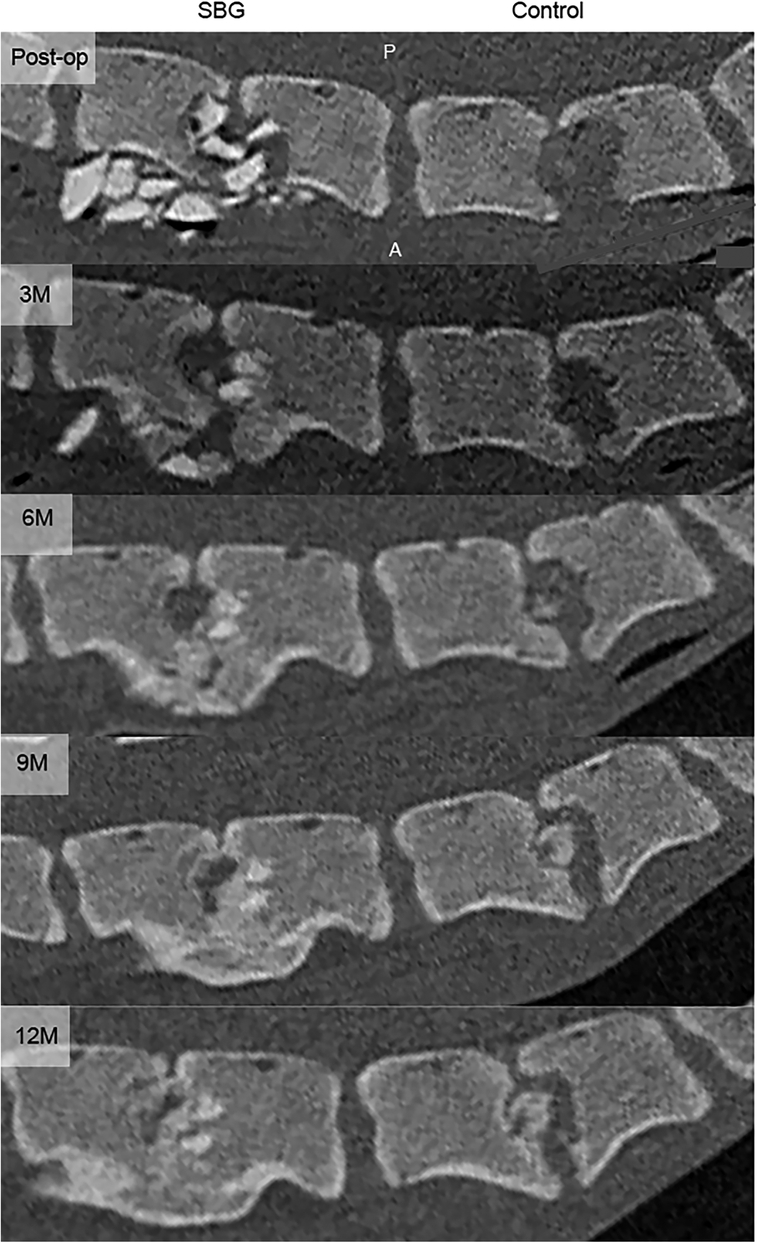
Chronological computed tomography images of a representative control and synthetic bone graft level. Midsagittal computed tomography images of synthetic bone graft (SBG) and control level of a representative animal over the study period. Fusion was evaluated through the entire intervertebral disc space as interbody bony fusion and fusion by spondylosis, defined as any continuous trabecular bony bridge within the disc space or outside the disc space, respectively. Interbody bony fusion was observed at three, six, nine, and 12 months postsurgery and fusion by spondylosis fusion by spondylosis at nine and 12 months for this animal. A indicates anterior; M, months postsurgery; *P,* posterior; postop, postoperative.

Increased IBF rates were observed at SBG levels compared with negative control levels at six and nine months postsurgery (Table 1 and Fig. 1; 4/4 SBG, 0/4 control; *P*=0.029). All SBG levels (4/4) were also fused by IBF at 12 months postsurgery in comparison with one control level (1/4). In contrast, no difference in fusion rate was observed when FbS was evaluated between the SBG and control levels (Table 2). All the pigs had a radiographically normal cervical vertebral column before surgery.

**TABLE 1:** Interbody Bony Fusion Rate

	3 mo	6 mo†	9 mo†	12 mo
SBG fusion rate	2/4	4/4	4/4	4/4
Control fusion rate	0/4	0/4	0/4	1/4

The number of levels, out of the total number of levels operated for the control and synthetic bone graft (SBG) groups, was evaluated by computed tomography as interbody bony fusion during the 12-month study period. Fusion rates were compared using the Fisher exact test for each timepoint.

† *P*=0.029.

Statistical significance was set at *P*<0.05.

**TABLE 2:** Fusion by Spondylosis Rate

	3 mo	6 mo	9 mo	12 mo
SBG fusion rate	1/4	2/4	4/4	4/4
Control fusion rate	0/4	0/4	1/4	1/4

Number of levels, out of the total number of levels operated for the control and synthetic bone graft (SBG) group, was evaluated by computed tomography as fusion by spondylosis during the 12-month study period. Fusion rates were compared using the Fisher exact test for each timepoint.

SBG indicates synthetic bone graft.

Statistical significance was set at *P*<0.05.

**Figure 2:**
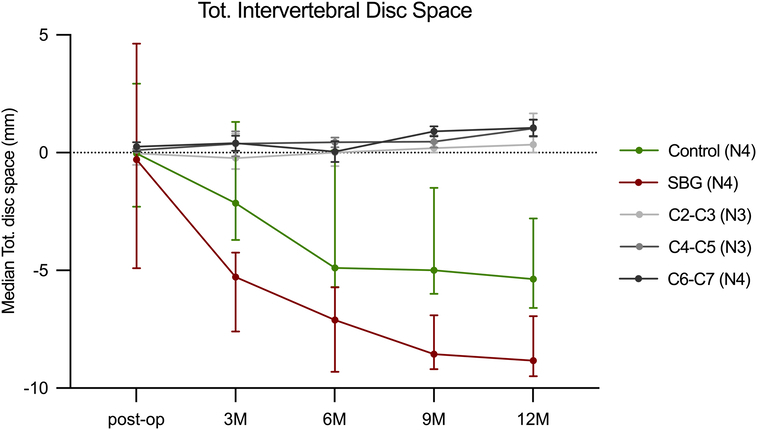
Median total intervertebral disc space. Median total (tot.) intervertebral disc space (mm) for all levels of the cervical spine was calculated as the sum of the minimum anterior, posterior, right, and left intervertebral disc spaces and normalized to baseline disc space for each level of the cervical spine at each timepoint (postop disc space—preop disc space). The error bars indicate the range. Multiple Wilcoxon tests corrected for multiple comparisons were used to compare the groups. C indicates cervical vertebral number; M, months postsurgery; N, number of animals; postop, postoperative; preop, preoperative; SBG, synthetic bone graft. Statistical significance was set at *P*<0.05.

Secondary CT results showed that the median total intervertebral disc space tended to decrease at SBG levels compared with that at control levels at nine and 12 months postsurgery (Fig. 2). Disc space did not tend to differ between levels before surgery and at unoperated disc levels over the 12-month study period (Fig. 2). Variable degrees of vertebral body sclerosis, lysis (low grades: Grade I–II, 1%–25%, and 26%–50%), and bone formation were detected at all time points during the study period (Table, SDC 4, Supplemental Digital Content 10, http://links.lww.com/BRS/C378; 5, Supplemental Digital Content 11, http://links.lww.com/BRS/C379; and 6, Supplemental Digital Content 12, http://links.lww.com/BRS/C380, vertebral body sclerosis, lysis, and bone formation). No differences were observed in R_ab_ between the SBG and control levels at any timepoint during the study period (Table 3).

**TABLE 3:** Spondylosis Anterioposterior Dimension Ratio

	3 mo	6 mo	9 mo	12 mo
SBG R_ab_	1.11 (0.72–1.45)	1.34 (1.17–1.55)	1.33 (1.21–1.48)	1.32 (1.19–1.45)
Control R_ab_	1.13 (0.99–1.18)	1.26 (1.02–1.43)	1.32 (0.99–1.64)	1.36 (1.05–1.60)

Comparison of spondylosis anterioposterior dimension ratio (R_ab_) calculated as spondylosis anterioposterior dimension 12 months after surgery (a) divided by disc space anterioposterior dimension before surgery (b) for synthetic bone graft (SBG) and control levels. R_ab_ reported as the median (range). R_ab_ was compared by using multiple Wilcoxon test corrected for multiple comparisons.

Statistical significance was set at *P*<0.05.

### Microcomputed tomography

**Figure 3:**
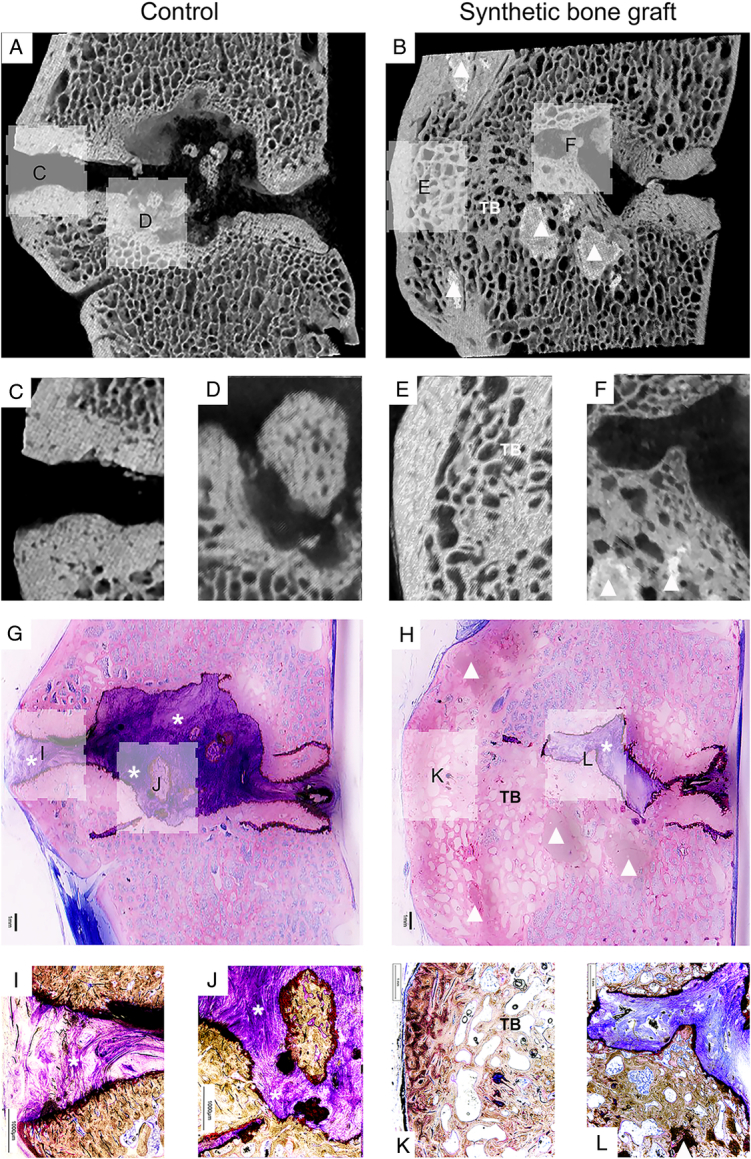
Microcomputed tomography and histopathology of a control and synthetic bone graft specimen. A representative midsagittal section of a control and synthetic bone graft level from the same animal was prepared using microcomputed tomography (μCT; A–F) and histopathology (G–L) 15 months postsurgery. Magnification of histopathologic (I–L) and corresponding μCT sections (C–F). The control group showed fibrous tissue (asterix) and bone islands but no trabecular bony bridging, and the synthetic bone graft level showed synthetic bone graft aggregates (triangle) and trabecular bony (TB) bridging. Histopathology sections were stained with modified paragon stain.

μCT acquisition 15 months postsurgery showed missing data for one of four animals; this animal was excluded from the qualitative μCT evaluation and was the same animal that showed fusion at the control level on CT evaluation. IBF and FbS were observed in all three remaining SBG levels (3/3) and in none of the three control levels (0/3). The SBG fusions were predominantly occupied by trabecular bony bridging, with a clear trabecular pattern (Fig. 3A–F).

### Histopathology

Histopathologic examination at 15 months postsurgery revealed IBF at all SBG levels (4/4) and none of the control levels (0/4). FbS was observed at all SBG levels (4/4) and only at one control level (1/4). SBG was considered to demonstrate minimal or no reaction to the tissue compared with the control, and degradation of SBG ranged from mild to strong (Table 4). Defects at the SBG level were partly filled with mature bone, whereas defects at the control level were filled with either or both fibrous tissue and necrotic material, sometimes mixed with cartilage and mature bone (Fig. 3G–L). The control fusion consisted of irregular trabeculae with active clast-like multinucleated cells, small marrow spaces, and less mature bone, compared with the more mature trabecular bone with only a few foci of immature woven bone in the SBG fusions (Figure, SDC7, Supplemental Digital Content 13, http://links.lww.com/BRS/C381, histology of control fusion). The bone marrow of SBG fusions consisted predominantly of adipose tissue with hematopoietic cells, only at the vertebral body interface. Aggregates of SBG, which were not completely resorbed by 15 months, were embedded in trabecular bone in three of four animals (Figure, SDC8, Supplemental Digital Content 14, http://links.lww.com/BRS/C382, histology of SBG) and appeared to be integrated in bone tissue in the fourth animal (Figure, SDC9, Supplemental Digital Content 15, http://links.lww.com/BRS/C383, histology of SBG). In three of the four animals, foci of macrophages and multinucleated giant cells were observed at the aggregate margins filled with granula resembling SBG material (Figure, SDC10, Supplemental Digital Content 16, http://links.lww.com/BRS/C384 and 11, Supplemental Digital Content 17, http://links.lww.com/BRS/C385, histology of SBG). Focally, osteogenic buds with clast-like cells and osteoblasts appeared to grow toward SBG aggregates (Figure, SDC12, Supplemental Digital Content 18, http://links.lww.com/BRS/C386, histology of SBG). No major inflammatory cell infiltrates or necrotic areas were observed at any of the levels evaluated.

**TABLE 4:** Histopathologic Tissue Reaction to Synthetic Bone Graft According to ISO 10992-6:2016

Cell type/response	SBG level				Control level			
	1	2	3	4	1	2	3	4
Polymorphonuclear cells	0	0	0	0	0	0	0	0
Lymphocytes	0	0	0	0	0	0	0	0
Plasma cells	0	0	0	0	0	0	0	0
Macrophages	0	0	0	0	0	0	0	0
Giant cells	(3+0+0)	(1+1+0)	(0+2+0)	0	0	0	0	0
Necrosis	0	0	(1+2+1)	0	(3+3+3)	0	(1+3+2)	0
Average subtotal×2	2	1,3	4	0	6	0	4	0
Neovascularization	0	0	0	0	0	0	0	0
Fibrosis	0	0	0	0	0	0	0	0
Fatty infiltrate	0	0	0	0	0	0	0	0
Subtotal×1	0	0	0	0	0	0	0	0
Total tissue response	2	1,3	4	0	6	0	4	0
Group total	7,3				10			
Average*	1,8 (-)				2,5=−0,7			
Conclusion	−0,7=0, Minimal or no reaction (0,0 to 2,9)							
Traumatic necrosis	0	0	0	0	0	0	0	0
Foreign debris (synthetic bone graft)	(3+3+0)	(2-2-0)	(0+3+1)	(0+1+0)	0	0	0	0
Degradation of synthetic bone graft	(3+3+1)	(3+3+0)	(0+3+3)	(NA+3 +NA)	NA	NA	NA	NA

Histopathologic evaluation of one left, central and right sagittal section for each animal at synthetic bone graft (SBG) and control level. Score 0 represent a score of 0 for all sections evaluated within that group. Scores in brackets represent the score of the left, central, and right sagittal section, respectively.

* A negative difference is recorded as zero in activity ranking in conclusion.

NA indicates not applicable; SBG, synthetic bone graft.

## DISCUSSION

In the present proof-of-concept study, by combining *in vivo* radiographic CT data and confirmatory *ex vivo* histologic fusion data in a noninstrumented porcine model of ACDF, a high spinal fusion success rate was demonstrated in the monetite-based SBG group in comparison to the negative control group. The significantly higher fusion rates observed at SBG levels as early as six and nine months postsurgery evaluated by CT are in line with clinical studies investigating cervical interbody fusion using beta-TCPs.^31,32^ These studies showed successful fusion at six months postsurgery after one-level or two-level discectomy and fusion. However, preclinical porcine lumbar fusion models investigating other bone graft substitutes show a variation in fusion success and time to interbody fusion. Bone marrow mesenchymal stem cells with a low-dose bone morphogenic protein showed a 100% fusion rate as early as three months postsurgery, whereas a tantalum ring with ABG showed a 68.2% fusion rate at six months.^33,34^ Early bony fusion is desirable, as bony fusion is a key factor in preventing excessive strain on stabilizing implants, which is considered the primary cause of screw loosening or pull-out.^35,36^ The higher fusion rate seen in the SBG group in the present study was further supported by the tendency toward decreased disc space seen in this group throughout the study period in comparison to the control group. Interestingly, all four SBG fusion masses originated from the intervertebral disc area, starting as an IBF before developing anteriorly to an FbS. In contrast, at the fused control level, the bridging bone originated anteriorly as an FbS and thereafter developed into an IBF. The different areas of fusion origin may be attributed to the capacity of SBG to initiate and augment bone formation.

Both μCT and histopathologic evaluation supported the results of higher fusion success at the SBG levels. In the three μCT specimens analyzed, IBF was confirmed to have a clear trabecular pattern. Histopathologic evaluation confirmed IBF at all four SBG levels and FbS at the one control level. However, histopathologic analysis did not detect IBF at the one-fused control level, whereas CT analysis did. This may be explained by the CT fusion evaluation being more accurate over the entire intervertebral space than the three sections used for histopathology. The finding of more mature bone in SBG fusions than in the one control fusion may indicate that SBG induces earlier bone remodeling and has a resorption rate matching the new bone formation rate, which is an important success factor for SBGs.^37,38^ However, further studies with longer follow-up periods are needed to evaluate the SBGs final transformation into bone, as aggregates of SBG were still observed at 15 months postsurgery, although no inflammatory cells around the aggregates were observed. Instead, mature bone with a histologic appearance similar to that of native bone was observed next to SBG aggregates, with minimal to moderate amounts of multinucleated giant cells and macrophages with intracellular SBG-like material. This is in line with other studies investigating a similar SBG.^25,39^ Osteogenic buds consisting of clast-like multinucleated cells and osteoblasts that may represent osteoclastic cutting cones were also observed in the histopathologic analysis. Shah and colleagues discovered that osteoclastic cutting cones play a passive but crucial role in the creation of irregularly shaped CaP islands when they investigated a similar multicomponent CaP formulation 12 months after implantation in an ovine animal model. This suggests a similar mechanism of bone healing and CaP degradation for the material used in the present study, although a more detailed cellular and molecular characterization is necessary to confirm this.

The results of this study need to be considered in light of its limitations. The small sample size limits the ability to detect significant differences in fusion rate as an effect of the SGB. However, according to the American Society for Testing and Materials F2884-21, a sample size of six to eight animals is sufficient for a proof-of-concept. The defect size and amount of SBG used were not standardized, which may have further limited the interpretation of the effect of SBG efficacy on fusion rates. In addition, a positive control comparing SBG to the gold standard ABG would provide important information on the clinical effectiveness of the SBG. Furthermore, the porcine cervical vertebral column differs in morphology and function from the human cervical spine, although it has been shown to be a reasonable surrogate for the human cervical spine.^33,34,40^

In conclusion, this proof-of-concept study provides preliminary evidence for the favorable performance of this novel Ca-PP-containing and β-TCP-containing monetite SBG as a stand-alone alternative to the negative control level in a clinically relevant porcine model of ACDF. By applying an array of strong assessment methods, including *in vivo* CT at relevant time points and confirmatory *ex vivo* histopathology at 15 months postsurgery, a high fusion rate throughout the study period was demonstrated, and high-quality bony fusion was confirmed. These findings support the premise that multicomponent monetite-based bone grafts may be effective bone graft substitutes for ACDF and justify a larger ongoing clinical investigation of these materials for use in spinal fusion.

Key Points

- This study preliminarily examined the effectiveness of a calcium pyrophosphate-containing and beta-tricalcium phosphate-containing monetite synthetic bone graft (SBG) in a porcine anterior cervical discectomy and fusion model compared with a negative control.
- Complete interbody bony fusion rates at six and nine months were significantly higher in the SBG group than in the negative control group.
- At 12-month follow-up, fusion was observed at all SBG levels and at one control level.
- High-quality bony fusion was confirmed in the SBG group at 15 months by histopathology.

## Supplementary Material

SUPPLEMENTARY MATERIAL
