# Supplementary material for: Novel Calcium Phosphate Promotes Interbody Bony Fusion in a Porcine Anterior Cervical Discectomy and Fusion Model
Source: Spine (Phila Pa 1976). 2024 Jan 12;49(17):1179–86. doi: 10.1097/BRS.0000000000004916 (PMC11319082; doi:10.1097/BRS.0000000000004916)
Supplement: SUPPLEMENTARY MATERIAL [file brs-49-1179-s001.pdf]

SDC Table 1: Experimental animals

Demographics of the experimental animals in the present study. Values presented as mean (standard deviation).

**Experimental animals**	
Animal species (strain):	Pig (Ellegaard Göttingen Minipigs, Denmark)
Gender:	Female
Number of animals (N):	4
Age:	24 (3,5) months
Body weight at start of study:	55 (5) kg
Body weight increase over the study period:	16.2%
Diet:	Standard mini-pig diet containing 1,01% calcium and 0,59% phosphorous, Special Diets Services, UK and hay
Housing:	Single housed in pens around 3-5m^2, within sight, smell and sound of one another
Environment enrichments:	Straw bedding, daily 10-15 min exercise in walkway and socialization with the other study-pigs in walkway through the pen bars
