# Supplementary material for: Novel Calcium Phosphate Promotes Interbody Bony Fusion in a Porcine Anterior Cervical Discectomy and Fusion Model
Source: Spine (Phila Pa 1976). 2024 Jan 12;49(17):1179–86. doi: 10.1097/BRS.0000000000004916 (PMC11319082; doi:10.1097/BRS.0000000000004916)
Supplement: SUPPLEMENTARY MATERIAL [file brs-49-1179-s002.pdf]

SDC Table 2: Anesthesia, analgesia and euthanasia of experimental animals

Drugs used for anesthesia, analgesia and euthanasia for the animals in the present study.

**Use**	**Drug (trade name), dose, administration**	**Manufacturer**
Induction:	Dexmedetomidine, (Dexdomitor), 0,025 mg/kg IM	Orion, Finland
	Zolazepam/Tiletamine (Zoletil), Zolazepam 2,5 mg/kg, Tiletamine 2,5 mg/kg IM	Virbac, Switzerland
	Butorphanol (Dolorex), 10mg/ml, 0,1 mg/kg IM	Intervet, Netherlands
Maintenance:	Sevoflurane (SevoFlo), 100%, inhelative	Zoetis, Finland
Peri-operative analgesia:	Dexmedetomidine (Dexdomitor), 4 mg/kg/h CRI	Orion, Finland
	or fentanyl (Fentadon), 7mcg/kg/h CRI	Dechra, Netherlands
Post-operative analgesia 3-5 days:	Buprenorphine (Temgesic) 0,03mg/kg IM	Indivior, Switzerland
	or transdermal fentanyl (Durogesic), 100 -150µg/h TD, ~2,4-3,6 mg/day	TD Janssen, Belgium
Post-operative analgesia 5 days:	Meloxicam (Metacam), 0,6-0,7 mg/kg PO	Boehringer Ingelheim, Germany
Sedation:	Zolazepam/Tiletamine (Zoletil), Zolazepam 2,5 mg/kg, Tiletamine 2,5 mg/kg IM	Virbac, Switzerland
	Butorphanol (Dolorex), 10mg/ml, 0,1 mg/kg IM	Intervet, Netherlands
Euthanasia:	Pentobarbital (Euthanimal Vet), 200mg/ml, 100mg/kg IC	VM Pharma, Netherlands
