# Supplementary material for: Novel Calcium Phosphate Promotes Interbody Bony Fusion in a Porcine Anterior Cervical Discectomy and Fusion Model
Source: Spine (Phila Pa 1976). 2024 Jan 12;49(17):1179–86. doi: 10.1097/BRS.0000000000004916 (PMC11319082; doi:10.1097/BRS.0000000000004916)
Supplement: SUPPLEMENTARY MATERIAL [file brs-49-1179-s003.pdf]

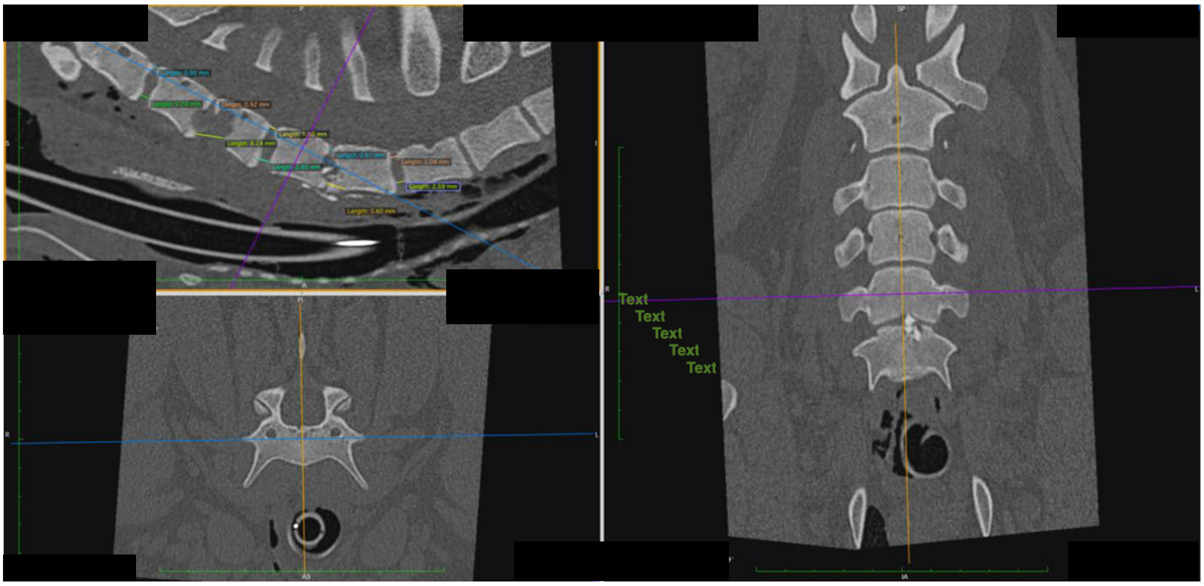

SDC Figure 1: Positioning of animals for cervical spine computer tomography measurements

Use 3D MPR of the Spine Helical 0.6 B70s series. If the computer asks you a ‘High Dynamic Values’ question when you do the 3D MPR then select ‘Clip’ (don’t select resample). Measurement of dorsal and ventral intervertebral disc space dimension. Align sagittal image so that it is mid sagittal in the dorsal plane image. Measure the lowest width of the dorsal half and the ventral half of each disc space. Minimum distance (use 0.1 mm unit) between the vertebrae (width of intervertebral disc space) in 4 locations, dorsal, ventral, right and left margins of the intervertebral disc space.
