# Supplementary material for: Novel Calcium Phosphate Promotes Interbody Bony Fusion in a Porcine Anterior Cervical Discectomy and Fusion Model
Source: Spine (Phila Pa 1976). 2024 Jan 12;49(17):1179–86. doi: 10.1097/BRS.0000000000004916 (PMC11319082; doi:10.1097/BRS.0000000000004916)
Supplement: SUPPLEMENTARY MATERIAL [file brs-49-1179-s006.pdf]

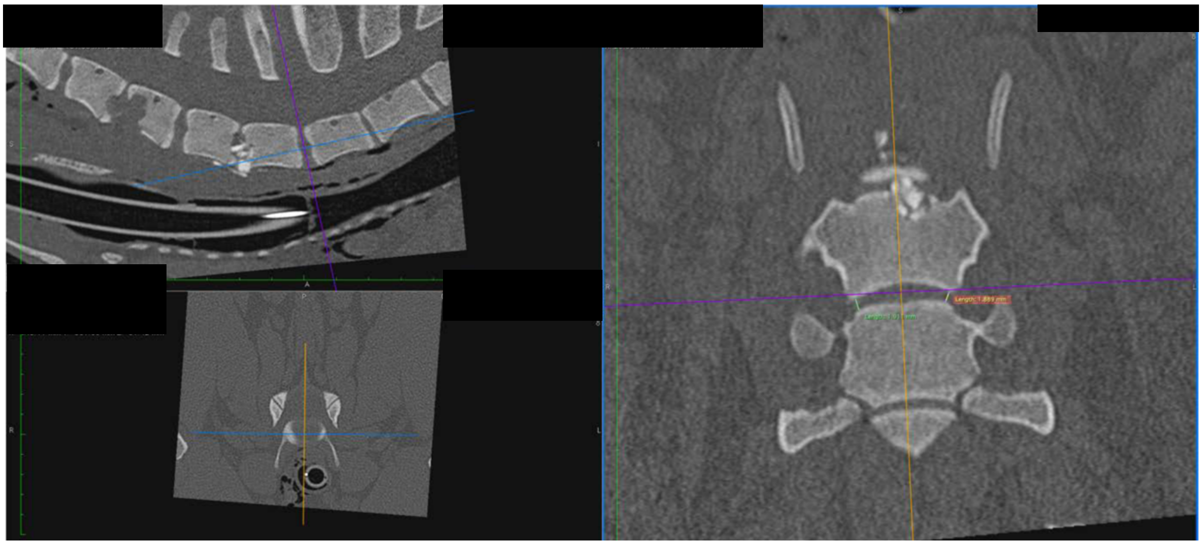

SDC Figure 4: Alignment of image when measuring non-operated level.

Alignment of the 3D MPR image to measure the lowest left and right width of C6-7 intervertebral disc space in a non-operated disc space. Mid sagittal plane, in the middle of the disc space with transverse plane parallel to the disc space.
