# Supplementary material for: Novel Calcium Phosphate Promotes Interbody Bony Fusion in a Porcine Anterior Cervical Discectomy and Fusion Model
Source: Spine (Phila Pa 1976). 2024 Jan 12;49(17):1179–86. doi: 10.1097/BRS.0000000000004916 (PMC11319082; doi:10.1097/BRS.0000000000004916)
Supplement: SUPPLEMENTARY MATERIAL [file brs-49-1179-s007.pdf]

SDC Figure 5: CT grading protocol

Pig name and journal number:

Radiologist name:

Examination date:

Date images graded:

Location of the control operated disc space:

Location of the synthetic bone graft operated disc space:

<u>Intervertebral disc space (3D MPR of the Spine Helical 0.6 B70s series)</u>

1. Minimum intervertebral disc space (use 0.01 mm unit) between the vertebrae (width of intervertebral disc space) in 4 locations, dorsal (posterior), ventral (anterior), right and left halves of the intervertebral disc space. Total intervertebral disc space was normalized to baseline for each level each timepoint (post-op disc space – pre-op disc space).

	Minimum width of disc space (0.01 mm)				
	Dorsal (posterior)	Ventral (anterior)	Right	Left	Tot. disc space, normalized to baseline
C2-3					
C3-4					
C4-5					
C5-6					
C6-7					

2. Defect size estimation (mm^3)

   3D MPR alignment at a sagittal plane centered over the defect, in the middle of the defect with transverse plane parallel to the disc space. Measure the longest distance of the defect (use 0.01 mm unit) length, width and height.

	Maximum defect size (0.01 mm)			
	Length	Width	Height	Volume of defect (mm^3)
SBG defect				
Control defect				

3. Fusion Evaluation

   - Interbody bony fusion present (Yes/No), evaluated through the entire intervertebral disc space, scroll back and forth through the disc space. Interbody fusion defined as: continuous trabecular bony bridge within the disc space.

   - Fusion by spondylosis present (Yes/No), defined as continuous trabecular bony bridge between two vertebras outside the disc space, scroll back and forth through the vertebral bodies. Use 3D MPR of the Spine Helical 0.6 B70s series.

	Interbody fusion Yes/No (Y/N)		Fusion by spondylosis Yes/No (Y/N)	
	Yes	No	Yes	No
C2-3				
C3-4				
C4-5				
C5-6				
C6-7				

4. Spondylosis anterioposterior dimension

Always do this measurement for the operated disc spaces, whether or not there is ventral (anterior) spondylosis (but also always answer the question ‘Ventral spondylosis Y/N). The

measurement must be done in the region of the intervertebral disc space (or fused intervertebral disc space) and is done on the same images used to measure the intervertebral disc space (so done in the mid-sagittal plane). Ratio is calculated according to formula below and reported reported as median (range). R_{ab}: spondylosis anterioposterior dimension ratio. (a): spondylosis anterioposterior dimension 12 months after surgery. (b): disc space anterioposterior dimension before surgery

$$R_{ab} = (a) / (b)$$

For non-operated disc spaces only do the measurement if spondylosis is present

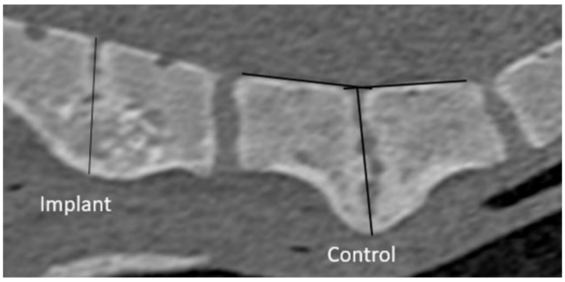

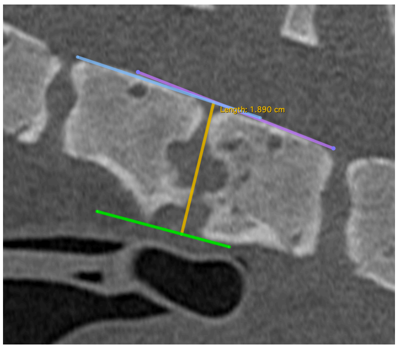

Measure the longest distance (use 0.01 mm unit) between bone formation on the dorsal (posterior) and ventral (anterior) aspects of the epiphyses of the vertebral bodies. If the bone formation is not fused/bridging then draw a line between the most ventral margins of the bone formation on each vertebrae and measure to the line.

	Control = C, Synthetic bone graft = S	Ventral (anterior) spondylosis Y/N	Size (0.01 mm)	R_{ab}
C2-3				
C3-4				
C4-5				
C5-6				

C6-7				

45

Vertebral body (use 3D MPR of the Spine Helical 0.6 B70s series)

Note that lysis, sclerosis and bone formation are determined by comparing to immediate postop CT images. The endplate defects present immediately after the surgery are not considered lysis, rather it is areas of hypoattenuation in the vertebrae that develop later. The gradings are done centered on the disc space and take into consideration the entire volume of the vertebrae (so scroll back and forth). The regions are 1) the caudal half and cranial half of the vertebral body with the disc space in the middle if there are changes (see vertical lines in picture below) then localize them to dorsal (posterior) (1) or ventral (anterior) (2) in the transverse and sagittal plane.

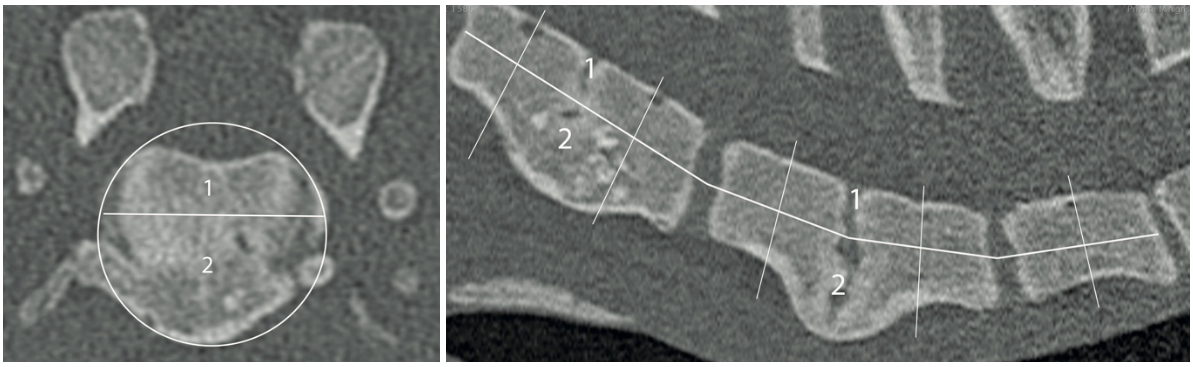

1. Sclerosis present Yes/No (Y/N), scroll back and forth through the disc space, Grade 0 = 0%, Grade 1 = 1-25%, Grade 2 = 26-50%, Grade 3 = 51-75%, Grade 4 = 75-99%, Grade 5 = 100%. For borderline grades always choose the lower grade. Localize any changes to dorsal (posterior) or ventral (anterior) half.
2. Lysis present Y/N, scroll back and forth through the disc space, Grade 0 = 0%, Grade 1 = 1-25%, Grade 2 = 26-50%, Grade 3 = 51-75%, Grade 4 = 75-99%, Grade 5 = 100%. For borderline grades always choose the lower grade. Localize any changes to dorsal (posterior) or ventral (anterior) half.

3. Bone formation within the intervertebral disc space Y/N. Localize any changes to dorsal (posterior) or ventral (anterior) half.

	Sclerosis			Lysis			Bone formation	
	Y/N	Grade	P/A	Y/N	Grade	P/A	Y/N	D/V
C2-3 Cr								
C2-3 Cd								
C3-4 Cr								
C3-4 Cd								
C4-5 Cr								
C4-5 Cd								
C5-6 Cr								
C5-6 Cd								
C6-7 Cr								
C6-7 Cd								

<u>Other findings</u>

Briefly describe any other lesions (not included in the grading) observed in the C2-C7 region (for example fractures, luxation/subluxation, angulation). When describing include location, attenuation, size, shape, number, margination information.
