# Supplementary material for: Novel Calcium Phosphate Promotes Interbody Bony Fusion in a Porcine Anterior Cervical Discectomy and Fusion Model
Source: Spine (Phila Pa 1976). 2024 Jan 12;49(17):1179–86. doi: 10.1097/BRS.0000000000004916 (PMC11319082; doi:10.1097/BRS.0000000000004916)
Supplement: SUPPLEMENTARY MATERIAL [file brs-49-1179-s008.pdf]

SDC Table 3: Semiquantitative scoring scheme according to ISO 10992-6:2016

Histopathological scoring scheme used to evaluate local tissue response to the synthetic bone graft 15 months post-surgery.

<table>
<tr><td colspan="6">Score</td></tr>
<tr><td>Cell type/response</td><td>0</td><td>1</td><td>2</td><td>3</td><td>4</td></tr>
<tr><td>Polymorphonucl. cells</td><td>0</td><td rowspan="4">Rare, 1 to 5/phf[a]</td><td rowspan="4">5-10/phf</td><td rowspan="5">Heavy infiltrate</td><td rowspan="4">Packed</td></tr>
<tr><td>Lymphocytes</td><td>0</td></tr>
<tr><td>Plasma cells</td><td>0</td></tr>
<tr><td>Macrophages</td><td>0</td></tr>
<tr><td>Giant cells</td><td>0</td><td>Rare, 1 to 2/phf</td><td>3-5/phf</td><td>Sheets</td></tr>
<tr><td>Necrosis</td><td>0</td><td>Minimal</td><td>Mild</td><td>Moderate</td><td>Severe</td></tr>
<tr><td colspan="6">[a] phf = per high-powered (400x) field.</td></tr>
<tr><td colspan="6">Score</td></tr>
<tr><td>Response</td><td>0</td><td>1</td><td>2</td><td>3</td><td>4</td></tr>
<tr><td>Neovascularization</td><td>0</td><td>Minimal capillary proliferation, focal, 1 to 3 buds</td><td>Groups of 4 to 7 capillaries with supporting fibroblastic structures</td><td>Broad band of capillaries with supporting fibroblastic structures</td><td>Extensive and of capillaries with supporting fibroblastic structures</td></tr>
<tr><td>Fibrosis</td><td>0</td><td>Narrow band</td><td>Moderately thick band</td><td>Thick band</td><td>Extensive band</td></tr>
<tr><td>Fatty infiltrate</td><td>0</td><td>Minimal amount of fat associated with fibrosis</td><td>Several layers of fat and fibrosis</td><td>Elongated and broad accumulation of fat cells around the synthetic bone graft site</td><td>Extensive fat completely surrounding the synthetic bone graft</td></tr>
<tr><td>Traumatic necrosis</td><td>None</td><td>Mild</td><td>Moderate</td><td>Obvious</td><td>Extensive area of necrosis</td></tr>
<tr><td>Foreign debris (synthetic bone graft)</td><td>None</td><td>Mild</td><td>Moderate</td><td>Obvious</td><td>Pronounced area of debris</td></tr>
<tr><td>Degradation of synthetic bone graft</td><td>None</td><td>Mild</td><td>Moderate</td><td>Strong</td><td>Heavy</td></tr>
<tr><td>Conclusion tissue reaction</td><td colspan="2">Minimal or no reaction (0,0 to 2,9)</td><td>Slight reaction (3,0 to 8,9)</td><td>Moderate reaction (9,0 to 15,0)</td><td>Severe reaction (15,1)</td></tr>
</table>
