# Supplementary material for: Novel Calcium Phosphate Promotes Interbody Bony Fusion in a Porcine Anterior Cervical Discectomy and Fusion Model
Source: Spine (Phila Pa 1976). 2024 Jan 12;49(17):1179–86. doi: 10.1097/BRS.0000000000004916 (PMC11319082; doi:10.1097/BRS.0000000000004916)
Supplement: SUPPLEMENTARY MATERIAL [file brs-49-1179-s009.pdf]

SDC Figure 6: Summary of necropsy report

At necropsy, none of the four animals had observable lesions in the tissues surrounding the surgery site. One of the animals had unilateral moderate osteoarthritis with focal erosion of the central part of the joint cartilage of the head of humerus, consistent with repaired lesions of osteochondrosis. Another had similar lesions bilaterally on the trochlea of humerus, while a third had mild discoloration and fibrillation in the same locations of both the condyle and the trochlea of humerus. In the fundus part of the ventricle of the first animal, several circular hyperemic foci, up to 10 mm diameter, were observed, but histological examination did not reveal pathological changes. The kidneys of all four animals were pale and had moderately increased texture. Histological examination showed mild to moderate diffuse interstitial fibrosis of both the renal medulla and cortex that might be consistent with previous episodes of contrast-induced acute kidney injury.
