# Supplementary material for: Novel Calcium Phosphate Promotes Interbody Bony Fusion in a Porcine Anterior Cervical Discectomy and Fusion Model
Source: Spine (Phila Pa 1976). 2024 Jan 12;49(17):1179–86. doi: 10.1097/BRS.0000000000004916 (PMC11319082; doi:10.1097/BRS.0000000000004916)
Supplement: SUPPLEMENTARY MATERIAL [file brs-49-1179-s010.pdf]

SDC Table 4: Vertebral body sclerosis

Vertebral body sclerosis evaluated by computed tomography graded from Grade 1 (1-25% sclerosis) to Grade 5 (100% sclerosis) at cranial half and caudal half of the vertebral body with the disc space in the middle, at control (C) and synthetic bone graft (SBG) levels, over 12 months. M=months post-surgery.

	3M		6M		9M		12M	
**Animal**	**SBG**	**C**	**SBG**	**C**	**SBG**	**C**	**SBG**	**C**
**1**	1	0	1	0	1	1	1	2
**2**	1	1	1	1	1	1	1	1
**3**	1	1	1	1	2	1	1	1
**4**	1	1	1	1	2	1	2	1
