# Supplementary material for: Novel Calcium Phosphate Promotes Interbody Bony Fusion in a Porcine Anterior Cervical Discectomy and Fusion Model
Source: Spine (Phila Pa 1976). 2024 Jan 12;49(17):1179–86. doi: 10.1097/BRS.0000000000004916 (PMC11319082; doi:10.1097/BRS.0000000000004916)
Supplement: SUPPLEMENTARY MATERIAL [file brs-49-1179-s011.pdf]

SDC Table 5: Vertebral body lysis

Vertebral body lysis evaluated by computed tomography graded from Grade 1 (1-25% lysis) to Grade 5 (100% lysis) at cranial half and caudal half of the vertebral body with the disc space in the middle, at control (C) and synthetic bone graft (SBG) levels, over 12 months. M=months post-surgery.

	3M		6M		9M		12M	
**Animal**	**SBG**	**C**	**SBG**	**C**	**SBG**	**C**	**SBG**	**C**
**1**	0	0	0	0	0	0	0	0
**2**	0	0	0	1	0	1	0	1
**3**	0	0	0	0	0	0	0	0
**4**	1	0	1	0	0	0	0	0
