# Supplementary material for: Novel Calcium Phosphate Promotes Interbody Bony Fusion in a Porcine Anterior Cervical Discectomy and Fusion Model
Source: Spine (Phila Pa 1976). 2024 Jan 12;49(17):1179–86. doi: 10.1097/BRS.0000000000004916 (PMC11319082; doi:10.1097/BRS.0000000000004916)
Supplement: SUPPLEMENTARY MATERIAL [file brs-49-1179-s012.pdf]

SDC Table 6. Bone formation within intervertebral disc space

Presence of bone formation within the intervertebral disc space at anterior or posterior half evaluated by computed tomography, for control (C) and synthetic bone graft (SBG) levels, over 12 months. M = months post-surgery. A = anterior half of disc space, P = posterior half of disc space

	3M		6M		9M		12M	
**Animal**	**SBG**	**C**	**SBG**	**C**	**SBG**	**C**	**SBG**	**C**
**1**	A	A	A/P	A/P	A/P	A/P	A/P	A/P
**2**	A/P	A	A	A	A/P	A	A/P	A
**3**	A/P	A	A/P	A/P	A/P	A/P	A	A/P
**4**	A/P	A	A	A	A/P	A	A/P	A/P
