# Supplementary material for: Novel Calcium Phosphate Promotes Interbody Bony Fusion in a Porcine Anterior Cervical Discectomy and Fusion Model
Source: Spine (Phila Pa 1976). 2024 Jan 12;49(17):1179–86. doi: 10.1097/BRS.0000000000004916 (PMC11319082; doi:10.1097/BRS.0000000000004916)
Supplement: SUPPLEMENTARY MATERIAL [file brs-49-1179-s013.pdf]

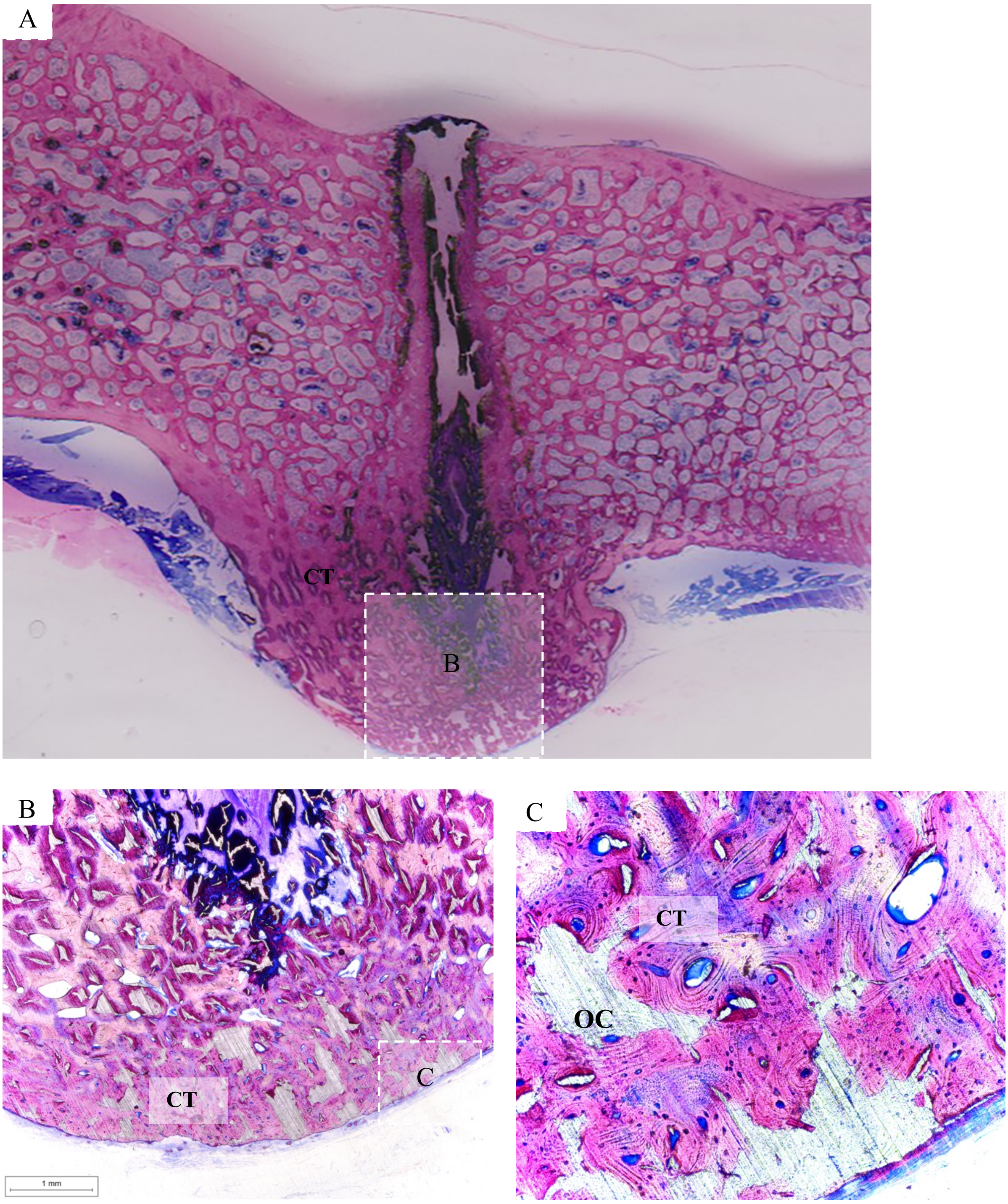

SDC Figure 7: Fusion by spondylosis in control sample.

Histopathological sagittal section of bony bridge at the one control level (A, B and C), where fusion had occurred, consisted of irregular and to some degree contorted trabeculae (CT) with active osteoclasts (OC), small marrow spaces and less mature bone.
