# Supplementary material for: Novel Calcium Phosphate Promotes Interbody Bony Fusion in a Porcine Anterior Cervical Discectomy and Fusion Model
Source: Spine (Phila Pa 1976). 2024 Jan 12;49(17):1179–86. doi: 10.1097/BRS.0000000000004916 (PMC11319082; doi:10.1097/BRS.0000000000004916)
Supplement: SUPPLEMENTARY MATERIAL [file brs-49-1179-s014.pdf]

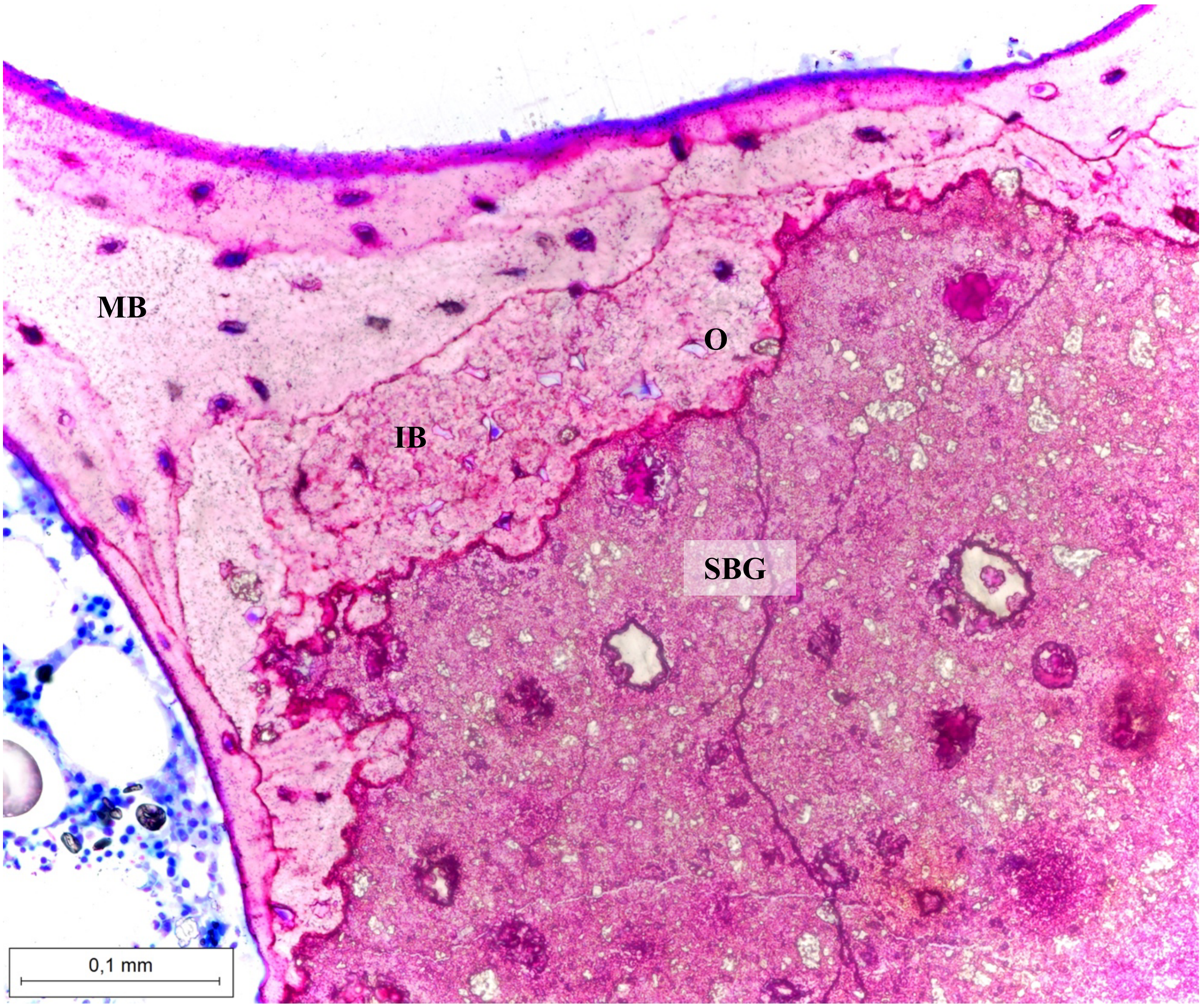

SDC Figure 8: Synthetic bone graft aggregate.

Histopathological section of synthetic bone graft level. Synthetic bone graft aggregate (SBG) were surrounded by a rim of carmoisine red material also presumed to represent osteoid (O). Similar material seemed to be present also within the aggregates without any obvious connection with bone, but confirmation of this observation would have required serial sectioning which hitherto not have been performed. The aggregates were variably surrounded by mature (MB) or immature bone (IB) or bone marrow.
