# Supplementary material for: Novel Calcium Phosphate Promotes Interbody Bony Fusion in a Porcine Anterior Cervical Discectomy and Fusion Model
Source: Spine (Phila Pa 1976). 2024 Jan 12;49(17):1179–86. doi: 10.1097/BRS.0000000000004916 (PMC11319082; doi:10.1097/BRS.0000000000004916)
Supplement: SUPPLEMENTARY MATERIAL [file brs-49-1179-s015.pdf]

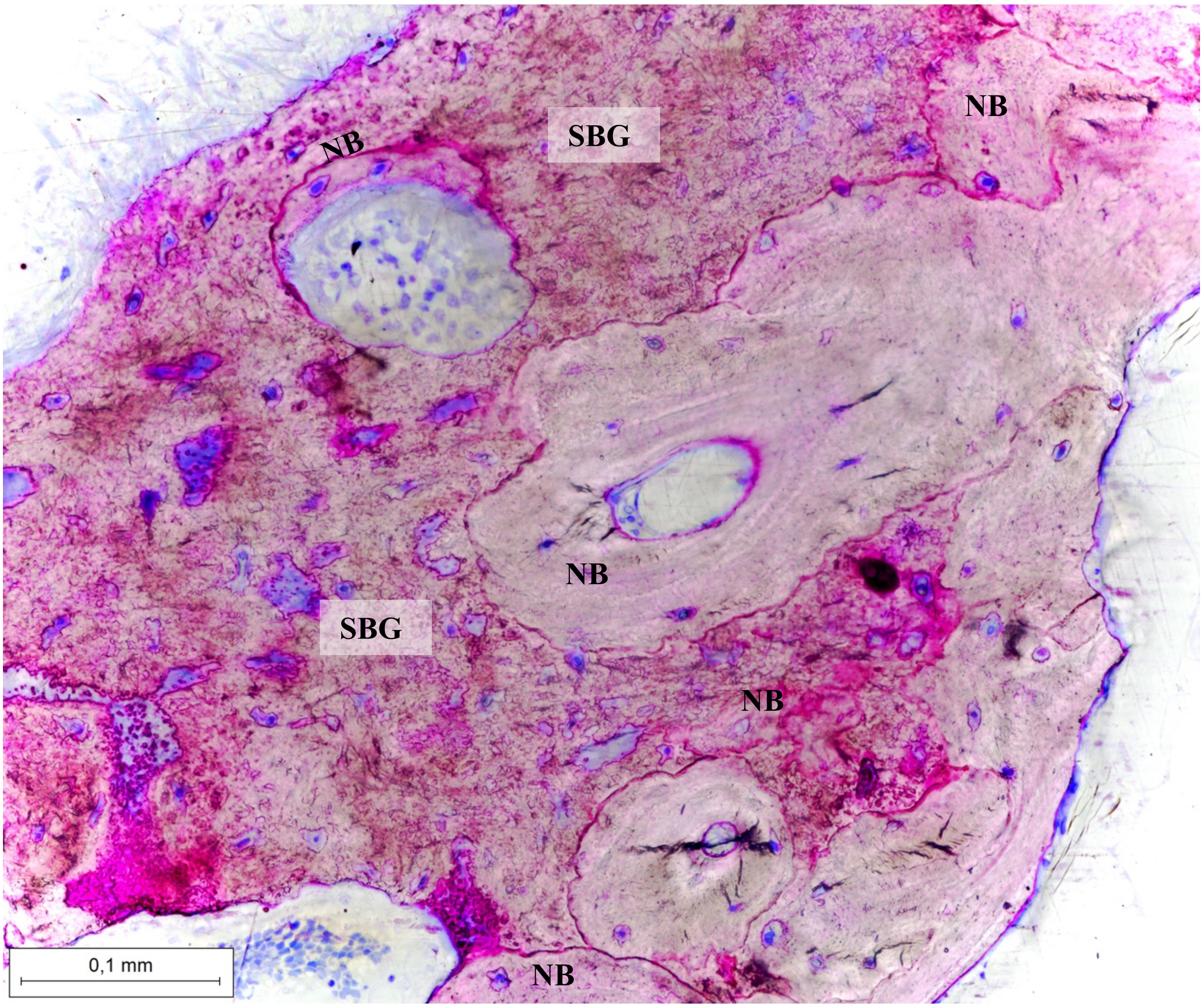

SDC Figure 9: Synthetic bone graft aggregate.

Histopathological section of synthetic bone graft level. Remains of synthetic bone graft (SBG) material were visible in one animal that appeared to have been integrated into the bone tissue, new bone (NB).
