# Supplementary material for: Novel Calcium Phosphate Promotes Interbody Bony Fusion in a Porcine Anterior Cervical Discectomy and Fusion Model
Source: Spine (Phila Pa 1976). 2024 Jan 12;49(17):1179–86. doi: 10.1097/BRS.0000000000004916 (PMC11319082; doi:10.1097/BRS.0000000000004916)
Supplement: SUPPLEMENTARY MATERIAL [file brs-49-1179-s016.pdf]

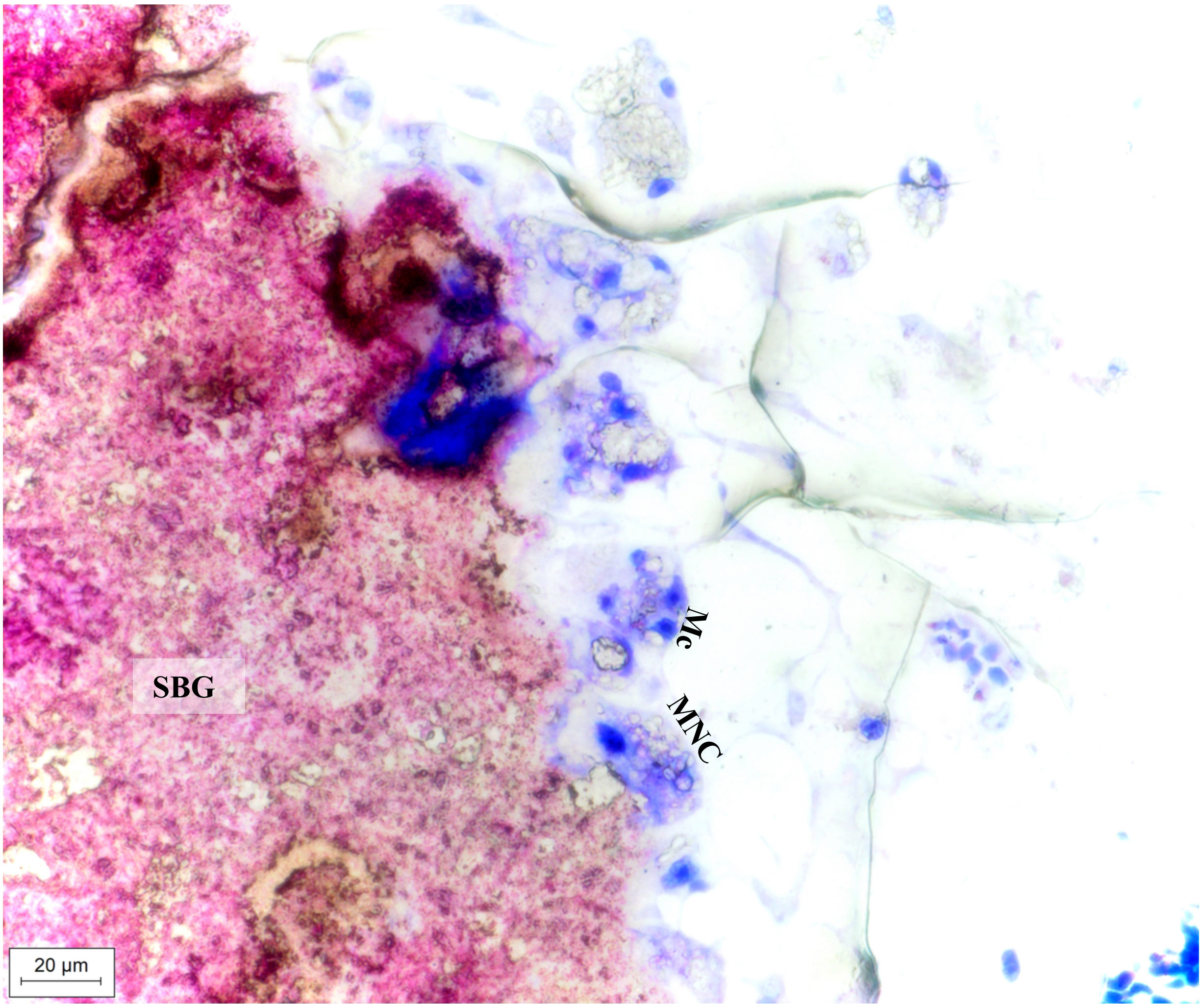

SDC Figure 10: Macrophages and multinucleated giant cells.

Histopathological section of synthetic bone graft level. Foci where the margins of the synthetic bone graft (SBG) aggregates were populated with macrophages (Mc) and multinucleated giant cells (MNC) filled with granular material resembling the synthetic bone graft was detected in three out of four animals.
