# Supplementary material for: Novel Calcium Phosphate Promotes Interbody Bony Fusion in a Porcine Anterior Cervical Discectomy and Fusion Model
Source: Spine (Phila Pa 1976). 2024 Jan 12;49(17):1179–86. doi: 10.1097/BRS.0000000000004916 (PMC11319082; doi:10.1097/BRS.0000000000004916)
Supplement: SUPPLEMENTARY MATERIAL [file brs-49-1179-s017.pdf]

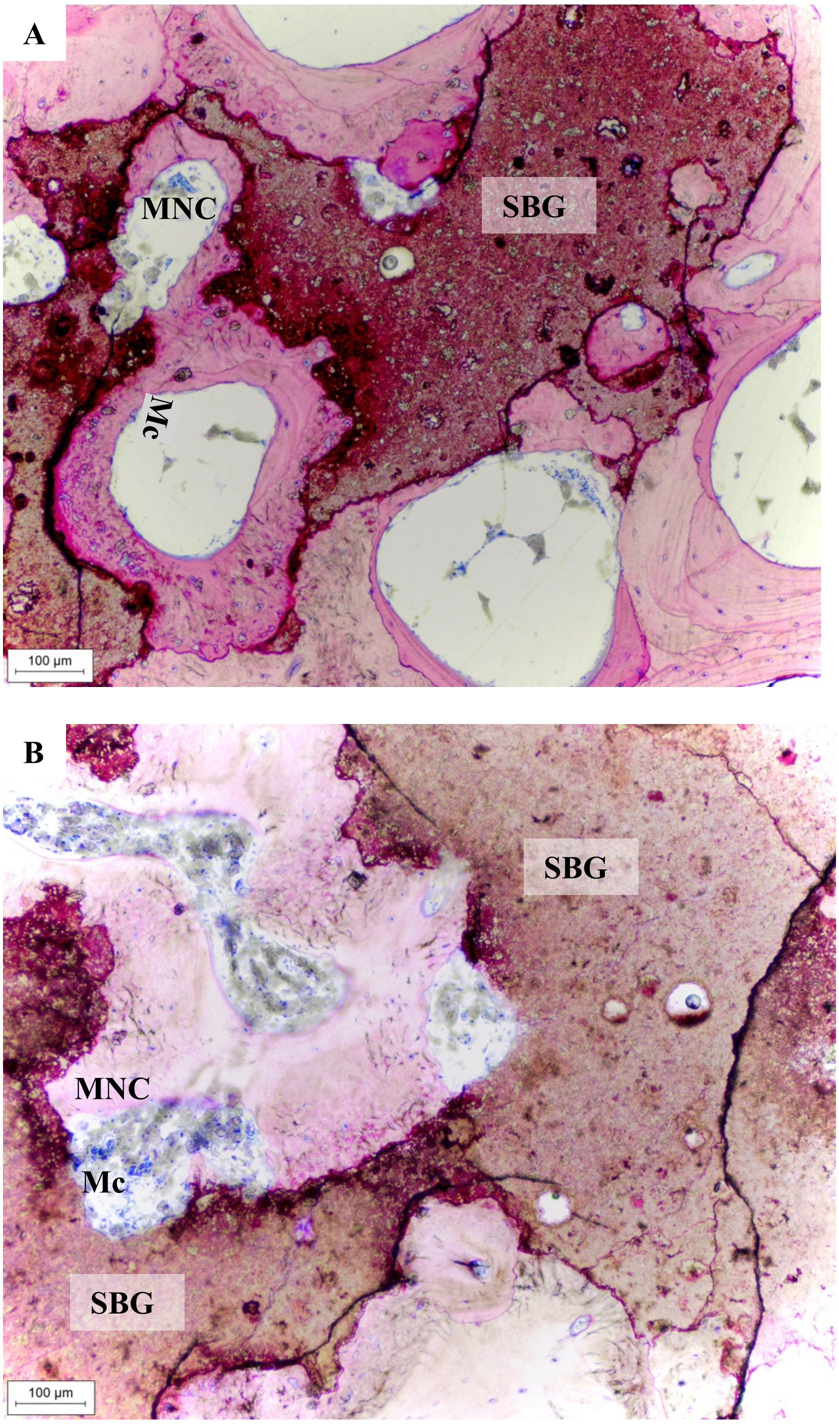

SDC Figure 11: Multinucleated giant cells.

Histopathological section of synthetic bone graft level (A and B). In a few foci, adjacent bone marrow to synthetic bone graft (SBG) aggregates was filled with multinucleated giant cells (MNC) and macrophages (Mc) filled with granular material resembling the synthetic bone graft.
