# Supplementary material for: Novel Calcium Phosphate Promotes Interbody Bony Fusion in a Porcine Anterior Cervical Discectomy and Fusion Model
Source: Spine (Phila Pa 1976). 2024 Jan 12;49(17):1179–86. doi: 10.1097/BRS.0000000000004916 (PMC11319082; doi:10.1097/BRS.0000000000004916)
Supplement: SUPPLEMENTARY MATERIAL [file brs-49-1179-s018.pdf]

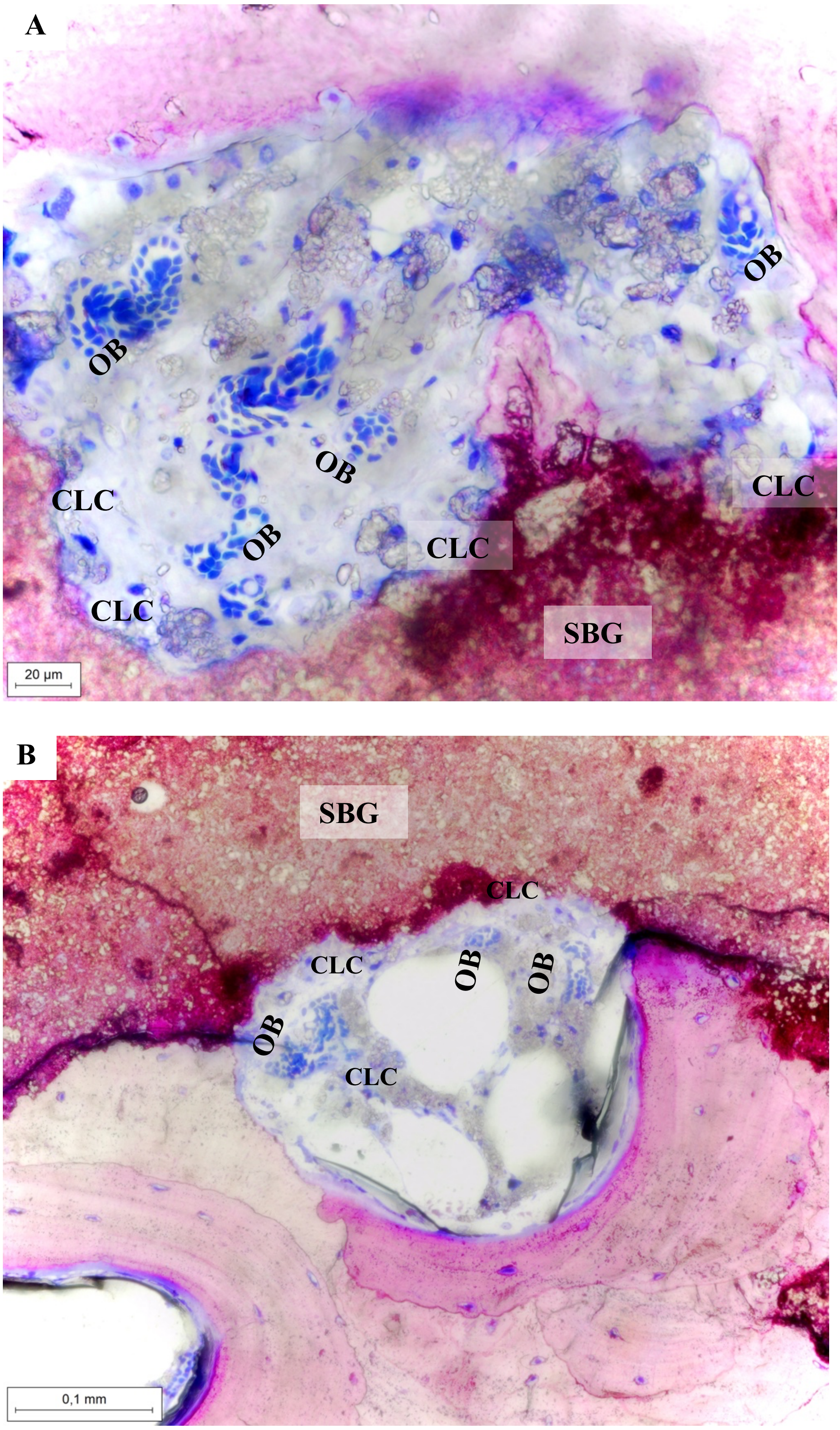

SDC Figure 12: Clast-like cells and osteoblasts.

Histopathological section of synthetic bone graft level (A and B). Osteogenic buds with clast-like multinucleated cells (CLC) and osteoblasts (OB) seemed to grow towards the aggregates of synthetic bone graft (SBG).
